# Supplementary material for: Master regulator genes and their impact on major diseases
Source: PeerJ. 2020 Oct 6;8:e9952. doi: 10.7717/peerj.9952 (PMC7546222; doi:10.7717/peerj.9952)
Supplement: Supplemental Information 1 [file peerj-08-9952-s001.docx]

**Supplementary Information**

Supplementary Table 1. Summary of tumor-related MRGs

| MRGs | Tumor diseasetype |
| --- | --- |
| T-Bet and GATA3 | T cell lymphoma (Matsumoto et al. 2010) |
| HNF | Pancreatic cancer, colon cancer (Janky et al. 2016; Kondratyeva et al. 2017; Odom et al. 2004) |
| GCM | Preeclampsia, parathyroid adenoma (Hashemolhosseini & Wegner 2004) |
| MITF | Melanoma (Levy et al. 2006) |
| Prox1 | Lymphangioendothelioma (Ke & Yang 2017) |
| pRb, p107, and p130 | Skin cancer (COSTA et al. 2013) |
| CDC45 | Multiple glioblastoma (GBM) and serous ovarian cancer (Gevaert & Plevritis 2012) |
| EWS/FLI | Ewing sarcoma /Bone malignancy (Tanner et al. 2017) |
| Sox2 | Many oncogenes of endoderm cancer, including the colon (Kuzmichev et al. 2012) |
| pRb | Retinoblastoma (Herwig & Strauss 1997; Rohde et al. 1996) |
| E2F8 | Metastatic prostate cancer (Lee et al. 2016) |
| heparanase | Myeloma (Purushothaman et al. 2008) |
| GRP78 | Pancreatic ductal adenocarcinoma(PDAC) (Gifford et al. 2016) |
| LEE | Intestinal epithelioma(LEE) (Brouwers et al. 2012) |
| 20 hox gene families | Skin cancer and breast cancer (Svingen & Tonissen 2003) |
| ESR1, FOXA1, GATA3，FOXM1, EZH2, MYBL2, ZNF695 | Human breast cancer (Li et al. 2015) |
| sox4 | Breast cancer (Parvani & Schiemann 2013) |
| Runx2 | Breast cancer (Wysokinski et al. 2015) |
| AMPK | Breast cancer (Brown & Simpson 2010) |
| TGF-β | Breast cancer (Fazilaty et al. 2013) |
| p53 | Breast cancer (Sharma et al. 2016) |
| MicroRNA | Breast cancer (Biagioni et al. 2012; Wright et al. 2010) |
| KDM4B | Breast cancer (Gaughan et al. 2013) |
| p16(INK4A） | Lymph node-negative breast cancer (Lanigan et al. 2015) |
| BACH1 | Breast cancer (Davudian et al. 2016; Liang et al. 2012) |
| Snail | Breast cancer (Takahashi et al. 2013) |
| HMGA1 | Breast cancer (Benecke & Eilebrecht 2015) |
| SATB1 | Breast cancer (Zheng 2008) |
| HSP90 | Breast cancer, lung cancer, colon cancer, prostate cancer, leukemia and skin cancer, etc. (Absarul et al. 2016; Zheng 2008) |
| Ddx5 and Ddx17 | Breast and prostate cancer (Samaan et al. 2014) |
| TRB3 | Breast cancer (Izrailit et al. 2013) |
| FGFR2 | Breast cancer (Fletcher et al. 2013) |
| AGTR2 | Breast cancer (Tovar et al. 2015) |
| HOX | Leukemia (McGonigle GJ 2008; Moore 2005; Rice & Licht 2007) |
| GATA | Leukemia (Bresnick et al. 2012) |
| CDX | Leukemia (Frohling et al. 2007) |
| RUNX1 | Leukemia (Ichikawa et al. 2004) |
| Pax | Leukemia (Medvedovic et al. 2011) |
| C/EBPα | Leukemia (Hankey et al. 2011) |
| PU.1 | Leukemia (Yang et al. 2012) |
| microRNAs | Leukemia (Huang J 2012) |
| CDC2 | Leukemia (Furukawa et al. 1995) |
| HSF1 | Leukemia (Li et al. 2016) |
| Ikaros | Leukemia (Dovat 2011) |
| pRB and INK4A/B | Leukemia (Tijchon et al. 2013) |
| Lin28 | Leukemia (Copley & Eaves 2013) |
| IKZF1 | Leukemia (Payne & Dovat 2011; Zhang & Bai 2015) |

**Supplementary** **References**

**Absarul H, Qamre A, Mohammad Zubair A, Esam IA, Khalid Hussain Wali S, Nisrin A, Gohar M, Mohammad Amjad K, and Mahmood R. 2016. Current Understanding of HSP90 as a Novel Therapeutic Target: An Emerging Approach for the Treatment of Cancer. *Current Pharmaceutical Design* 22:2947-2959.**

**Benecke AG, and Eilebrecht S. 2015. RNA-Mediated Regulation of HMGA1 Function. *Biomolecules* 5:943-957.**

**Biagioni F, Bossel Ben-Moshe N, Fontemaggi G, Canu V, Mori F, Antoniani B, Di Benedetto A, Santoro R, Germoni S, De Angelis F, Cambria A, Avraham R, Grasso G, Strano S, Muti P, Mottolese M, Yarden Y, Domany E, and Blandino G. 2012. miR-10b*, a master inhibitor of the cell cycle, is down-regulated in human breast tumours. *EMBO Molecular Medicine* 4:1214-1229.**

**Bresnick EH, Katsumura KR, Lee H-Y, Johnson KD, and Perkins AS. 2012. Master regulatory GATA transcription factors: mechanistic principles and emerging links to hematologic malignancies. *Nucleic acids research* 40:5819-5831.**

**Brouwers E, Ma I, and Thomas NA. 2012. Dual temporal transcription activation mechanisms control cesT expression in enteropathogenic Escherichia coli. *Microbiology* 158:2246-2261.**

**Brown KA, and Simpson ER. 2010. Obesity and Breast Cancer: Progress to Understanding the Relationship. *Cancer Research* 70:4.**

**Copley MR, and Eaves CJ. 2013. Developmental changes in hematopoietic stem cell properties. *Experimental & molecular medicine* 45:e55-e55.**

**COSTA C, Paramio J, and SANTOS M. 2013. Skin Tumors Rb(eing) Uncovered. *Frontiers in Oncology* 3.**

**Davudian S, Mansoori B, Shajari N, Mohammadi A, and Baradaran B. 2016. BACH1, the master regulator gene: A novel candidate target for cancer therapy. *Gene* 588:30-37.**

**Dovat S. 2011. Ikaros in hematopoiesis and leukemia. *World journal of biological chemistry* 2:105-107.**

**Fazilaty H, Gardaneh M, Bahrami T, Salmaninejad A, and Behnam B. 2013. Crosstalk between breast cancer stem cells and metastatic niche: emerging molecular metastasis pathway? *Tumor Biology* 34:2019-2030.**

**Fletcher MNC, Castro MAA, Wang X, de Santiago I, O'Reilly M, Chin S-F, Rueda OM, Caldas C, Ponder BAJ, Markowetz F, and Meyer KB. 2013. Master regulators of FGFR2 signalling and breast cancer risk. *Nature communications* 4:2464-2464.**

**Frohling S, Scholl C, Bansal D, and Huntly BJP. 2007. HOX Gene Regulation in Acute Myeloid Leukemia: CDX Marks the Spot? *Cell Cycle* 6:2241-2245.**

**Furukawa Y, Terui Y, Sakoe K, Ohta M, Kitagawa S, Miura Y, and Saito M. 1995. Over-expression and amplification of the CDC2 gene in leukaemia cells. *British Journal of Haematology* 90:94-99.**

**Gaughan L, Stockley J, Coffey K, O'Neill D, Jones DL, Wade M, Wright J, Moore M, Tse S, Rogerson L, and Robson CN. 2013. KDM4B is a master regulator of the estrogen receptor signalling cascade. *Nucleic acids research* 41:6892-6904.**

**Gevaert O, and Plevritis S. 2012. IDENTIFYING MASTER REGULATORS OF CANCER AND THEIR DOWNSTREAM TARGETS BY INTEGRATING GENOMIC AND EPIGENOMIC FEATURES. *Biocomputing 2013*: WORLD SCIENTIFIC, 123-134.**

**Gifford JB, Huang W, Zeleniak AE, Hindoyan A, Wu H, Donahue TR, and Hill R. 2016. Expression of GRP78, Master Regulator of the Unfolded Protein Response, Increases Chemoresistance in Pancreatic Ductal Adenocarcinoma. *Molecular Cancer Therapeutics* 15:1043-1052.**

**Hankey W, Silver M, Sun BSH, Zibello T, Berliner N, and Khanna-Gupta A. 2011. Differential effects of sumoylation on the activities of CCAAT enhancer binding protein alpha (C/EBPα) p42 versus p30 may contribute in part, to aberrant C/EBPα activity in acute leukemias. *Hematology reports* 3:e5-e5.**

**Hashemolhosseini S, and Wegner M. 2004. Impacts of a new transcription factor family: mammalian GCM proteins in health and disease. *The Journal of cell biology* 166:765-768.**

**Herwig S, and Strauss M. 1997. The Retinoblastoma Protein: A Master Regulator of Cell Cycle, Differentiation and Apoptosis. *European Journal of Biochemistry* 246:581-601.**

**Huang J MY. 2012. Role of microRNAs in leukemia stem cells. *Front Biosci (Schol Ed)* 4:799-809.**

**Ichikawa M, Asai T, Chiba S, Kurokawa M, and Ogawa S. 2004. Runx1/AML-1 Ranks as a Master Regulator of Adult Hematopoiesis. *Cell Cycle* 3:720-722.**

**Izrailit J, Berman HK, Datti A, Wrana JL, and Reedijk M. 2013. High throughput kinase inhibitor screens reveal TRB3 and MAPK-ERK/TGFβ pathways as fundamental Notch regulators in breast cancer. *Proceedings of the National Academy of Sciences of the United States of America* 110:1714-1719.**

**Janky Rs, Binda MM, Allemeersch J, Van den broeck A, Govaere O, Swinnen JV, Roskams T, Aerts S, and Topal B. 2016. Prognostic relevance of molecular subtypes and master regulators in pancreatic ductal adenocarcinoma. *BMC Cancer* 16:632.**

**Ke ZY, and Yang SJ. 2017. [Role of master transcriptional factor Prox-1 in lymphatic endothelial differentiation of Kaposiform hemangioendothelioma]. *Zhonghua bing li xue za zhi = Chinese journal of pathology* 46:176-181.**

**Kondratyeva LG, Chernov IP, Zinovyeva MV, Kopantzev EP, and Sverdlov ED. 2017. Expression of master regulatory genes of embryonic development in pancreatic tumors. *Doklady Biochemistry and Biophysics* 475:250-252.**

**Kuzmichev Andrey N, Kim S-K, D’Alessio Ana C, Chenoweth Josh G, Wittko Ina M, Campanati L, and McKay Ronald D. 2012. Sox2 Acts through Sox21 to Regulate Transcription in Pluripotent and Differentiated Cells. *Current Biology* 22:1705-1710.**

**Lanigan F, Brien GL, Fan Y, Madden SF, Jerman E, Maratha A, Aloraifi F, Hokamp K, Dunne EJ, Lohan AJ, Flanagan L, Garbe JC, Stampfer MR, Fridberg M, Jirstrom K, Quinn CM, Loftus B, Gallagher WM, Geraghty J, and Bracken AP. 2015. Delineating transcriptional networks of prognostic gene signatures refines treatment recommendations for lymph node-negative breast cancer patients. *The FEBS Journal* 282:3455-3473.**

**Lee S, Park YR, Kim S-H, Park E-J, Kang MJ, So I, Chun JN, and Jeon J-H. 2016. Geraniol suppresses prostate cancer growth through down-regulation of E2F8. *Cancer medicine* 5:2899-2908.**

**Levy C, Khaled M, and Fisher DE. 2006. MITF: master regulator of melanocyte development and melanoma oncogene. *Trends in Molecular Medicine* 12:406-414.**

**Li G, Song Y, Zhang Y, Wang H, and Xie J. 2016. miR-34b Targets HSF1 to Suppress Cell Survival in Acute Myeloid Leukemia. *Oncology Research Featuring Preclinical and Clinical Cancer Therapeutics* 24:109-116.**

**Li R, Campos J, and Iida J. 2015. A Gene Regulatory Program in Human Breast Cancer. *Genetics* 201:1341-1348.**

**Liang Y, Wu H, Lei R, Chong RA, Wei Y, Lu X, Tagkopoulos I, Kung S-Y, Yang Q, Hu G, and Kang Y. 2012. Transcriptional network analysis identifies BACH1 as a master regulator of breast cancer bone metastasis. *The Journal of biological chemistry* 287:33533-33544.**

**Matsumoto Y, Horiike S, Ohshiro M, Yamamoto M, Sasaki N, Tsutsumi Y, Kobayashi T, Shimizu D, Uchiyama H, Kuroda J, Nomura K, Shimazaki C, and Taniwaki M. 2010. Expression of Master Regulators of Helper T-Cell Differentiation in Peripheral T-Cell Lymphoma, Not Otherwise Specified, by Immunohistochemical Analysis. *American Journal of Clinical Pathology* 133:281-290.**

**McGonigle GJ LT, Thompson A. 2008. Grappling with the HOX network in hematopoiesis and leukemia. *Front Biosci* 13:297-308.**

**Medvedovic J, Ebert A, Tagoh H, and Busslinger M. 2011. Chapter 5 - Pax5: A Master Regulator of B Cell Development and Leukemogenesis. In: Alt FW, ed. *Advances in Immunology*: Academic Press, 179-206.**

**Moore MAS. 2005. Converging pathways in leukemogenesis and stem cell self-renewal. *Experimental Hematology* 33:719-737.**

**Odom DT, Zizlsperger N, Gordon DB, Bell GW, Rinaldi NJ, Murray HL, Volkert TL, Schreiber J, Rolfe PA, Gifford DK, Fraenkel E, Bell GI, and Young RA. 2004. Control of Pancreas and Liver Gene Expression by HNF Transcription Factors. *Science (New York, NY)* 303:1378-1381.**

**Parvani JG, and Schiemann WP. 2013. Sox4, EMT programs, and the metastatic progression of breast cancers: mastering the masters of EMT. *Breast cancer research : BCR* 15:R72-R72.**

**Payne KJ, and Dovat S. 2011. Ikaros and tumor suppression in acute lymphoblastic leukemia. *Critical reviews in oncogenesis* 16:3-12.**

**Purushothaman A, Chen L, Yang Y, and Sanderson RD. 2008. Heparanase stimulation of protease expression implicates it as a master regulator of the aggressive tumor phenotype in myeloma. *The Journal of biological chemistry* 283:32628-32636.**

**Rice KL, and Licht JD. 2007. HOX deregulation in acute myeloid leukemia. *The Journal of clinical investigation* 117:865-868.**

**Rohde M, Warthoe P, Gjetting T, Lukas J, Bartek J, and Strauss M. 1996. The retinoblastoma protein modulates expression of genes coding for diverse classes of proteins including components of the extracellular matrix. *Oncogene* 12:2393-2401.**

**Samaan S, Tranchevent L-C, Dardenne E, Polay Espinoza M, Zonta E, Germann S, Gratadou L, Dutertre M, and Auboeuf D. 2014. The Ddx5 and Ddx17 RNA helicases are cornerstones in the complex regulatory array of steroid hormone-signaling pathways. *Nucleic acids research* 42:2197-2207.**

**Sharma S, Patnaik PK, Aronov S, and Kulshreshtha R. 2016. ApoptomiRs of Breast Cancer: Basics to Clinics. *Frontiers in genetics* 7:175-175.**

**Svingen T, and Tonissen KF. 2003. Altered HOX Gene Expression in Human Skin and Breast Cancer Cells. *Cancer Biology & Therapy* 2:518-523.**

**Takahashi R-u, Takeshita F, Honma K, Ono M, Kato K, and Ochiya T. 2013. Ribophorin II regulates breast tumor initiation and metastasis through the functional suppression of GSK3β. *Scientific reports* 3:2474-2474.**

**Tanner JM, Bensard C, Wei P, Krah NM, Schell JC, Gardiner J, Schiffman J, Lessnick SL, and Rutter J. 2017. EWS/FLI is a Master Regulator of Metabolic Reprogramming in Ewing Sarcoma. *Molecular Cancer Research* 15:1517.**

**Tijchon E, Havinga J, van Leeuwen FN, and Scheijen B. 2013. B-lineage transcription factors and cooperating gene lesions required for leukemia development. *Leukemia* 27:541-552.**

**Tovar H, García-Herrera R, Espinal-Enríquez J, and Hernández-Lemus E. 2015. Transcriptional master regulator analysis in breast cancer genetic networks. *Computational Biology and Chemistry* 59:67-77.**

**Voorhoeve PM. 2010. MicroRNAs: Oncogenes, tumor suppressors or master regulators of cancer heterogeneity? *Biochimica et Biophysica Acta (BBA) - Reviews on Cancer* 1805:72-86.**

**Wright JA, Richer JK, and Goodall GJ. 2010. microRNAs and EMT in Mammary Cells and Breast Cancer. *Journal of Mammary Gland Biology and Neoplasia* 15:213-223.**

**Wysokinski D, Blasiak J, and Pawlowska E. 2015. Role of RUNX2 in Breast Carcinogenesis. *International journal of molecular sciences* 16:20969-20993.**

**Yang H, Liang H, Yan J-s, Tao R, Hao S-g, and Ma L-y. 2012. Down-regulation of hematopoiesis master regulator PU.1 via aberrant methylation in chronic myeloid leukemia. *International Journal of Hematology* 96:65-73.**

**Zhang H-Y, and Bai H. 2015. [Effect of Ikaros in B Cell Acute Lymphoblastic Leukemia]. *Zhongguo shi yan xue ye xue za zhi* 23:1194-1198.**

**Zheng J. 2008. Is SATB1 a Master Regulator in Breast Cancer Growth and Metastasis? *Women's Health* 4:329-332.**
